# Genome-Wide Identification and Expression Analysis of the TCP Gene Family Related to Developmental and Abiotic Stress in Ginger

**DOI:** 10.3390/plants12193389

**Published:** 2023-09-26

**Authors:** Yajun Jiang, Dongzhu Jiang, Maoqin Xia, Min Gong, Hui Li, Haitao Xing, Xuedong Zhu, Hong-Lei Li

**Affiliations:** 1 College of Landscape Architecture and Life Science, Chongqing University of Arts and Sciences, Chongqing 402160, China; jiangyajun228@163.com (Y.J.); jiangdongzhu11@163.com (D.J.); xiamq@cqwu.edu.cn (M.X.); gongmin1999@163.com (M.G.); lihui121797@163.com (H.L.); 2 Yudongnan Academy of Agricultural Sciences, Chongqing 408000, China

**Keywords:** ginger, TCP genes, growth, expression pattern, abiotic stress

## Abstract

Ginger (*Zingiber officinale Roscoe*), a widely consumed edible and medicinal plant, possesses significant nutritional and economic value. Abiotic stresses such as drought and low temperatures can impact the growth and development of ginger. The plant-specific transcription factor Teosinte branched1/cycloidea/proliferating cell factor (*TCP*) has progressively been identified in various plants for its role in regulating plant growth and development as well as conferring resistance to abiotic stresses. However, limited information on the *TCP* family is available in ginger. In this study, we identified 20 *TCP* members in the ginger genome, which were randomly distributed across 9 chromosomes. Based on phylogenetic analysis, these ginger *TCP* were classified into two subfamilies: Class I (PCF) and Class II (CIN, CYC/TB). The classification of the identified ginger *TCPs* was supported by a multi-species phylogenetic tree and motif structure analysis, suggesting that the amplification of the ginger *TCP* gene family occurred prior to the differentiation of angiosperms. The promoter region of ginger *TCP* genes was found to contain numerous cis-acting elements associated with plant growth, development, and abiotic stress response. Among these elements, the stress response element, anaerobic induction, and MYB binding site play a dominant role in drought responsiveness. Additionally, expression pattern analysis revealed variations in the expression of ginger *TCP* gene among different tissues and in response to diverse abiotic stresses (drought, low temperature, heat, and salt). Our research offers a thorough examination of *TCP* members within the ginger plant. This analysis greatly contributes to the understanding of how *TCP* genes regulate tissue development and response to stress, opening up new avenues for further exploration in this field.

## 1. Introduction

Transcription factors are proteins that play a key role in plant growth and development by binding to specific gene promoter or enhancer regions [1]. The *TCP* gene family is a class of plant-specific transcription factors involved in plant growth and development [2,3]. Since its first discovery in 1999 [4], it has gained considerable attention and favor from researchers. The *TCP* gene family is widely distributed in different plant species, from lower plants such as moss and algae, to higher plants such as Arabidopsis, maize, rice, and watermelon, in which *TCP* genes have been identified [5,6,7]. The name of the *TCP* transcription factor is derived from a combination of four closely related but unrelated genes that encode proteins from three different species. These genes include Teosinte branched1 (TB1) from maize, which regulates apical dominance and inflorescence development [8,9]. The cycloidea (CYC) gene from snapdragon is a major factor controlling flower symmetry development [10,11]. And the proliferating cell factor 1 and 2 (PCF1 and PCF2) genes from rice are mainly involved in lateral organ development and binding to the promoter of proliferating cell nuclear antigen genes [12]. All members of the *TCP* family contain a basic helix–loop–helix (bHLH) atypical secondary structure, consisting of about 60 amino acid residues. This bHLH domain is a conserved structure required for DNA binding and protein interaction, and it plays an important role in regulating downstream gene expression in plant biological processes [13,14]. Based on the analysis of the conserved domain sequence homology, *TCP* can be divided into two subfamilies, Class I and Class II. Class I is also known as the PCF class, while Class II further splits into the CIN and CYC/TB1 branches [6,13]. The most significant difference between the two subfamilies is that Class II TCP proteins have four additional amino acid residues in their domain compared to Class I.

As the identification of plant genomes progresses, research on the *TCP* gene family becomes more in-depth. Increasing evidence shows that *TCP* genes play a crucial regulatory role in many biological processes of plant growth and development. For example, they are involved in branching development, leaf shape formation, seed germination, flower organ development, regulation of circadian rhythms in plants, modulation of plant hormone signaling pathways, and response to biotic and abiotic stresses [3,15,16,17,18,19,20,21,22,23]. Based on the expression patterns of *OsPCF1*, *OsPCF2*, and *AtTCP20* in meristematic tissues, it has been found that Class I TCP members primarily promote plant cell proliferation, differentiation, and leaf growth [12,24]. In Arabidopsis, both *AtTCP14* and *AtTCP15* are involved in regulating plant height [25]. Additionally, these two genes stimulate the response of leaves and flowers to cytokinins and promote seed germination in the presence of gibberellins (GA) [19]. *AtTCP9* and *AtTCP20* regulate leaf senescence through the jasmonic acid signaling pathway [26]. Clearly, from the observations mentioned above, it can be inferred that Class I members have redundant functions in regulating plant growth and development.

Class II *TCP* genes are further divided into two subclasses, CIN and CYC/TB1. In Arabidopsis, *TCP2/TCP3/TCP4/TCP5/TCP10/TCP13/TCP17/TCP24* belong to the CIN subclass, while *TCP1/TCP12/TCP18* belong to the CYC/TB1 subclass. CIN-type *TCP* genes possess recognition sequences for microRNA319, and their transcription is regulated by miRNA. CYC/TB1-type TCP proteins have a region rich in glutamic acid–cysteine–glutamic acid near the C-terminus, and their lateral sides often contain serine and valine [4,27]. CIN-type *TCP* genes are involved in regulating leaf shape formation in plants. In Arabidopsis, the quadruple mutants of members such as *AtTCP2*, *AtTCP3*, *AtTCP4*, *AtTCP10*, and *AtTCP24* upregulate plant cyclin and cell division-related genes, resulting in strongly serrated leaf phenotype [16,28]. CYC/TB1-type *TCP* genes regulate axillary meristem development and branching. *TB1* genes with branching regulatory functions have also been found in maize and rice. *AtTCP12* and *AtTCP18* are closely related to ZmTB1, and their single mutants can suppress the expression of these two genes, leading to increased branching [29].

Additionally, TCP transcription factors play a crucial role in plant responses to various abiotic stresses, including low temperature, drought, and salt stress. When plants are subjected to abiotic stress, their growth and development are severely affected. In rice (*Oryza sativa*), *OsTCP19* is upregulated under drought, salt, and cold stress, suggesting its involvement in stress tolerance. Additionally, downregulating the expression of *OsPCF6* and *OsTCP21* enhances rice’s tolerance to cold stress by altering its ability to scavenge reactive oxygen species [30,31]. Binding of *OsPCF2* with the OsNHX1 promoter improves salt and drought tolerance [32]. In Arabidopsis, *TCP20* interacts with NIN-like protein 6 (NLP6) and NLP7 to regulate signal transduction and nitrate assimilation, thereby controlling root growth [33]. In other plants, such as moso bamboo, *PeTCP10* enhances salt stress tolerance [34].

Ginger (*Zingiber officinale*), a perennial herb belonging to the Zingiberaceae family and Zingiber genus, features yellow-green flowers and pungent rhizomes. It is extensively cultivated in China, primarily through vegetative propagation. Both the aerial and underground parts of ginger hold significant utilization value. The subterranean stem possesses medicinal and dietary characteristics, containing various natural compounds that offer substantial nutritional and medical benefits. Additionally, ginger exhibits favorable traits such as high yield per unit area and economic profitability, making it a promising crop worth promoting [35,36]. Ginger often experiences a decrease in yield and quality when subjected to abiotic factors such as drought and low temperatures during its growth. However, there is currently limited research on *TCP* in ginger, despite the fact that *TCP* plays a crucial role in various abiotic stresses in model plants. In our previous research, we sequenced and assembled the ginger genome, laying the foundation for identifying *TCP* genes in ginger [37]. In this study, we identified *TCP* members from the ginger genome. Through bioinformatics analysis, we analyzed the characteristics of *TCP* family members, chromosome localization, gene structure, promoter cis-acting elements, phylogenetic relationships, tissue expression patterns, as well as duplication events and collinearity analysis. Additionally, we analyzed the expression profiles of *TCP* under low temperature, drought, heat, and salt stress treatments. The results of this study provide a theoretical basis for further investigating the potential functions and regulatory mechanisms of *ZoTCP* genes in response to abiotic stress.

## 2. Results

### 2.1. Identification and Characterization of TCP Gene Family in Ginger

The standard *TCP* transcription factor family (PF03634) was retrieved from the Pfam database. Using hmmer 3.0 software and establishing a hidden Markov model, a search was conducted in the ginger genome database, yielding a total of 20 ginger *TCP* genes. These genes were subsequently named *ZoTCP1—ZoTCP20* based on their respective chromosomal positions (Table 1). The physicochemical properties of the protein encoded by each ginger *TCP* gene, including the number of amino acids, isoelectric point, and relative molecular mass, were analyzed. The amino acid length ranged from 211 to 713 aa, with a relative molecular weight between 23.49 and 78.70 kD. The isoelectric points varied from 5.77 to 9.38. Notably, 55% (11) of *ZoTCP* genes exhibited a PI > 7.00, indicating an abundance of basic amino acids (Table 1).

### 2.2. Chromosomal Localization Analysis of TCP Gene in Ginger

**Figure 1:**
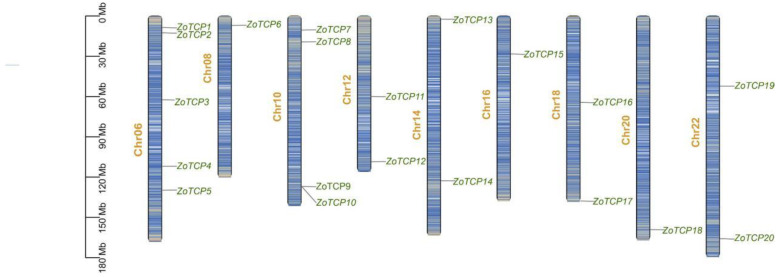
Chromosome distribution of *TCP* family genes in *Zingiber officinale*.

Using the available genome information of ginger, a chromosome mapping map was generated (Figure 1). Analysis of the chromosome localization information for the *ZoTCP* gene family revealed an uneven distribution pattern among the 20 *TCP* genes in ginger. Random distribution was observed on nine chromosomes, including Chr.06 to Chr.22, with varying numbers of *TCP* genes ranging from one to five. Remarkably, the Chr.06 chromosome harbored the highest number of *TCP* genes, with a total of five. Conversely, the Chr.08, Chr.16, and Chr.20 chromosomes each contained only one *TCP* gene. Additionally, Chr.12, Chr.14, Chr.16, and Chr.22 accommodated two *TCP* genes, while Chr.10 possessed four *TCP* genes. Notably, the positions of *ZoTCP9* and *ZoTCP10* on Chr.10 were found to be highly similar, suggesting a potential tandem replication event in the evolution of the *ZoTCP* gene family. This event may have contributed to the evolutionary dynamics and diversification within the *ZoTCP* gene family.

### 2.3. Evolutionary Analysis of Ginger TCP Gene Family

**Figure 2:**
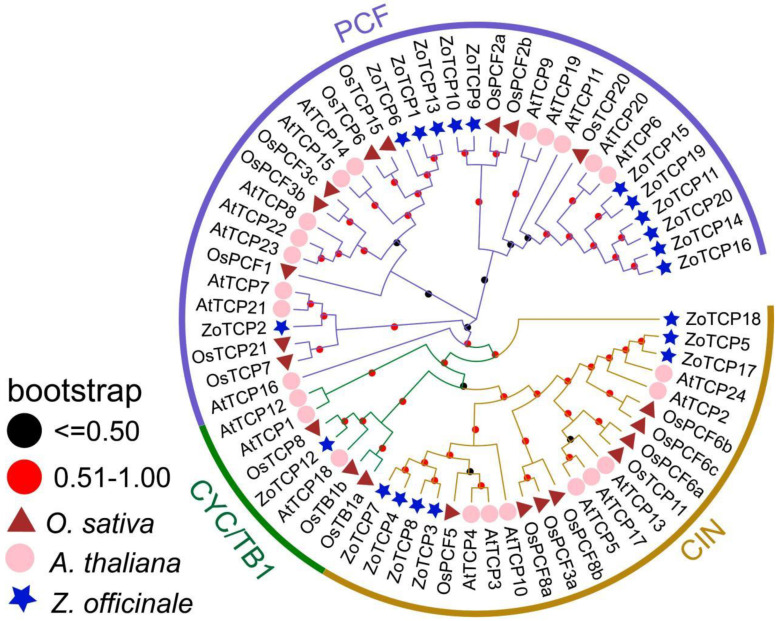
A rootless phylogenetic tree was constructed based on TCP proteins of ginger (*Zingiber officinale*), *Arabidopsis thaliana,* and rice (*Oryza sativa*). The phylogenetic tree was divided into PCF, CIN, and CYC/TB.

A total of 65 full protein sequences, including 24 *AtTCP*, 21 *OsTCP*, and 20 *ZoTCP*, were used in the analysis. In general, the topologies and support values for the TCP family inferred using maximum likelihood (ML) and neighbor-joining (N-J) are congruent (Figure 2 and Figure S1). Based on the phylogenetic tree and clustering results of Arabidopsis and rice *TCP* genes, the *ZoTCP* genes can be divided into two main subclasses: Class I and Class II. Among the 20 *ZoTCPs*, twelve were classified as Class I (PCF), while the remaining eight were classified as Class II. Furthermore, within Class II, two subgroups were identified: CIN and CYC/TB1. Interestingly, the CYC/TB1 subgroup contained only one gene, *ZoTCP12* (Figure 2). At the same time, we utilized the maximum likelihood method to construct a phylogenetic tree in order to validate the phylogenetic tree generated by the neighbor-joining method. The results obtained from the maximum likelihood method (Supplementary Material File S2: Figure S1) demonstrate consistency with the neighbor-joining method.

**Figure 3:**
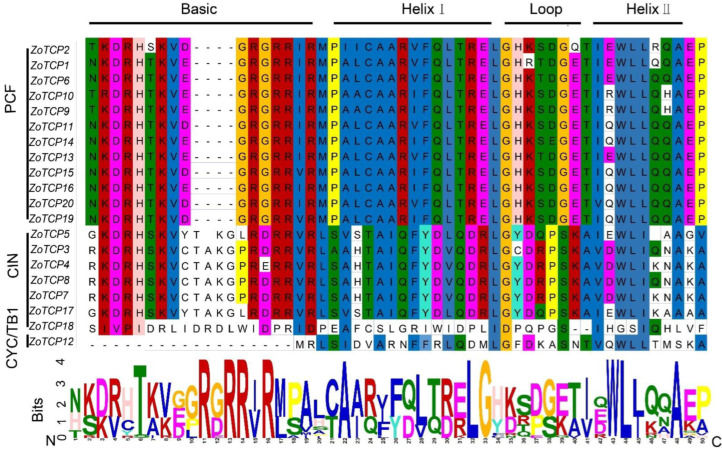
The multiple sequence alignment of ZoTCP protein was divided into an alkaline region basic and Helix I–Loop–Helix II.

To gain further insight into the evolutionary relationship and structural characteristics of the ginger *TCP* gene family, a conserved domain sequence alignment analysis was conducted using the 20 identified *ZoTCP* full protein sequences. The results (Figure 3) revealed that all 20 *ZoTCP* members possessed a conserved *TCP* domain consisting of 54 amino acid residues. This domain comprised a basic region at the N-terminus and a helix–loop–helix motif at the C-terminus. Notably, the basic region in Class I had four fewer amino acid residues compared to Class II. This structural feature is consistent with the *TCP* structure observed in other plant species, indicating a high degree of conservation during the evolution of *TCP* genes across different plants. Additionally, the Helix I–Loop-Helix II region exhibited greater conservation in Class I compared to Class II. Interestingly, among the *ZoTCPs* members, only *ZoTCP12* belongs to the CYC/TB1 group, and its basic region lacks any amino acid residues. This suggests that *ZoTCP12* may be a pseudogene with functional defects.

### 2.4. ZoTCP Gene Structure and Conserved Motif Analysis

**Figure 4:**
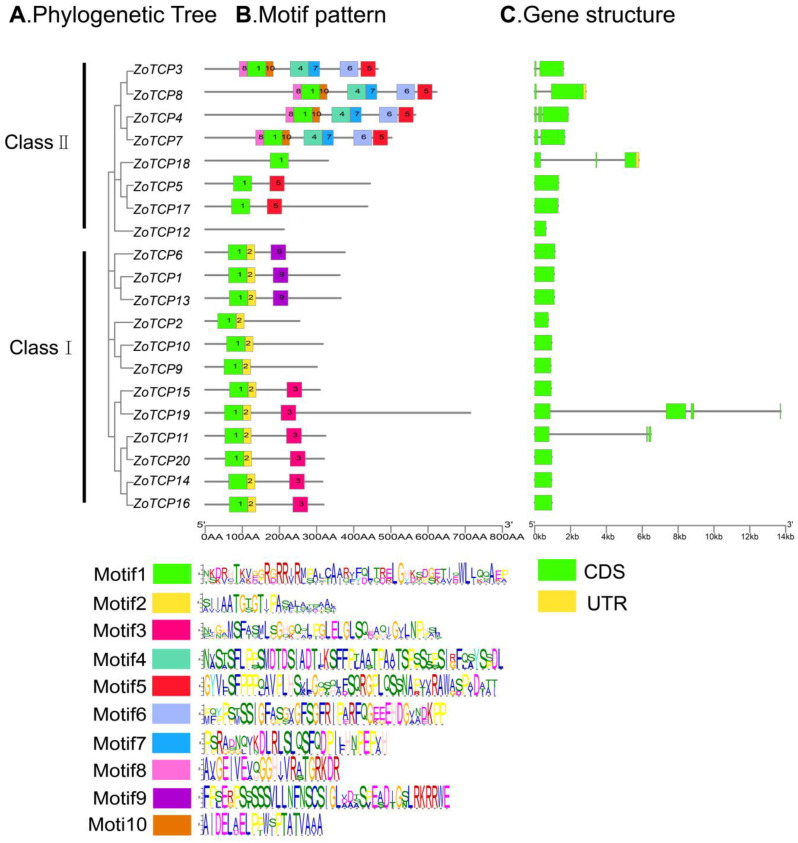
Phylogenetic relationship, gene structure, and conserved motif structure of ZoTCP protein. (**A**) Phylogenetic tree was constructed based on ZoTCP protein. (**B**) The motif structure of ZoTCP protein contains 10 Motifs (Supplementary Material File S3: Figure S2). (**C**) Exon–intron structure.

To gain insights into the diversity and evolutionary relationship of ginger *TCP* genes, we conducted a comparative analysis of *ZoTCP* gene sequences to determine their structural characteristics. The results (Figure 4B) revealed the presence of 10 conserved motifs among the 20 *ZoTCP* genes studied. Notably, Motif1 was detected in 19 out of the 20 *ZoTCP* genes, suggesting its high conservation and potential importance in performing essential functions within the *ZoTCP* gene family. On the other hand, *ZoTCP12* did not possess any motifs (Figure 4B), indicating a structural deletion resulting from gene mutation during evolution. The observed branch patterns in the phylogenetic tree (Figure 4A) corresponded closely with the presence of conserved motifs within *ZoTCP* family members. For instance, members of the PCF class, namely *ZoTCP15*, *ZoTCP19*, *ZoTCP11*, *ZoTCP20*, *ZoTCP14*, and *ZoTCP16*, shared Motif1, Motif2, and Motif3. Similarly, *ZoTCP6*, *ZoTCP1*, and *ZoTCP13* exhibited Motif9, while CIN-like *ZoTCP3*, *ZoTCP4*, *ZoTCP8*, and *ZoTCP7* displayed a consistent number and type of conserved motifs, which was also the largest among the subfamilies. These structural features reflect the functional distinctions among each subfamily and are consistent with the corresponding phylogenetic relationships. Thus, the conserved motifs provide additional evidence supporting the classification and diverse functions of the ginger *TCP* gene subfamilies.

The analysis of gene structures revealed that the 20 *ZoTCP* genes exhibited relatively simple structures (Figure 4C). Among them, only 2 out of the 20 *ZoTCP* genes (10%) contained both coding regions (CDS) and untranslated regions (UTR), while the remaining 18 genes (90%) consisted solely of coding regions. Notably, *ZoTCP19* possessed three introns, while *ZoTCP4*, *ZoTCP18*, and *ZoTCP11* contained two introns. The rest of the genes had a simpler structure, consisting of either one or no introns. When comparing the findings from the phylogenetic tree and gene structure analysis, it was observed that members within the same subgroup exhibited similarities in terms of CDS composition and intron presence, indicating a certain level of conservation. This suggests that members within the same subgroup may share similar biological functions. However, overall, the gene structure of *ZoTCP* genes is relatively simple.

### 2.5. Evolutionary Analysis of ZoTCP Gene

**Figure 5:**
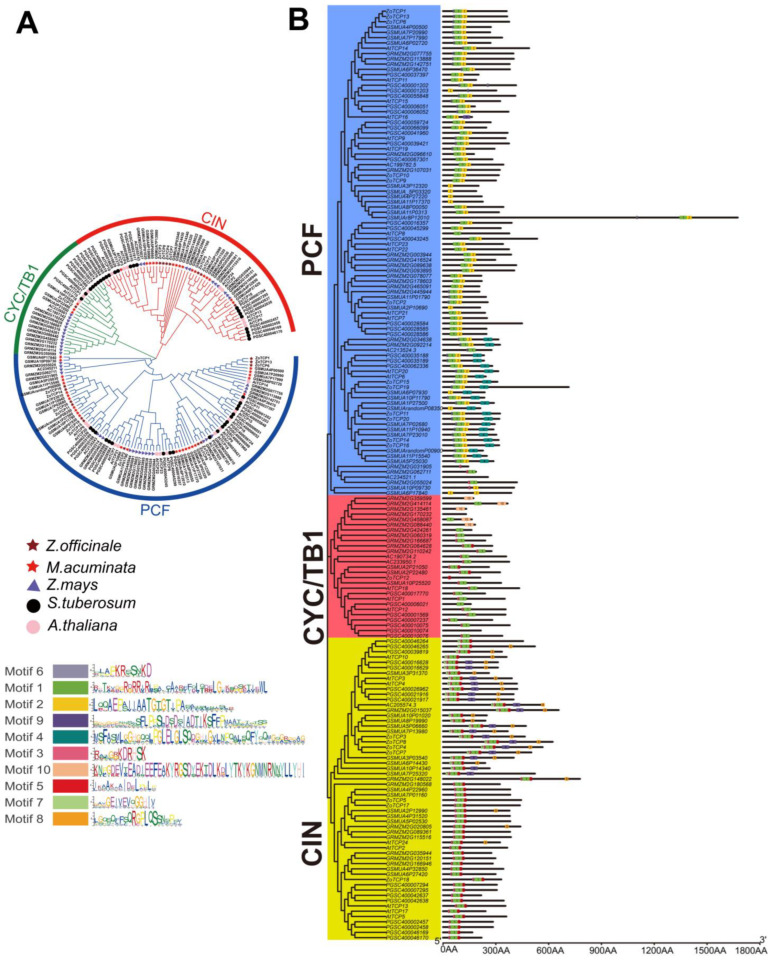
The phylogenetic relationship and conserved protein motif structure of TCP proteins in *Z. officinale*, *S. tuberosum*, *A. thaliana*, *Z. mays*, and *M. acuminata*. (**A**) According to the rootless phylogenetic tree constructed by TCP proteins of five species, it is divided into PCF, CYC/TB1, and CIN—three subgroups. (**B**) TCP protein motif (Supplementary Material File S3: Figure S3) composition structure of 5 species, including 10 motifs.

To investigate the evolutionary relationship of *TCP* genes, we conducted a comprehensive analysis that included the construction of a phylogenetic tree (Figure 5A) using *TCP* full sequence proteins from *ZoTCP*, as well as two dicotyledonous plants (Arabidopsis thaliana and potato) and two monocotyledonous plants (maize and banana). Additionally, we analyzed the conserved motifs present in their TCP complete protein sequences (Figure 5B). Following the classification approach used for Arabidopsis, *TCP* members in each species were divided into two subfamilies: Class I (PCF) and Class II (CIN and CYC/TB1). Based on the phylogenetic tree (Figure 5A), it was observed that ginger, banana, and maize often clustered together within each subfamily. Furthermore, most members from Arabidopsis and potato were grouped within the same branch. This clustering pattern may be attributed to their distinct plant orders, suggesting a potential relationship between order classification and *TCP* gene grouping. Interestingly, both subfamilies were represented in *TCP* members across all five species studied, indicating a relatively recent differentiation of these species from a common ancestor. Analysis of TCP protein motifs revealed the presence of ten major conserved motifs among the five species (Figure 5B). Notably, Motif1 was found in almost all *TCP* members, which aligns with the findings from *ZoTCP* analysis and highlights its highly conserved nature. Furthermore, members within the same subfamily exhibited similar compositions of main motifs, indicating a highly conserved motif structure within subfamilies.

### 2.6. Cis-Acting Element Analysis

**Figure 6:**
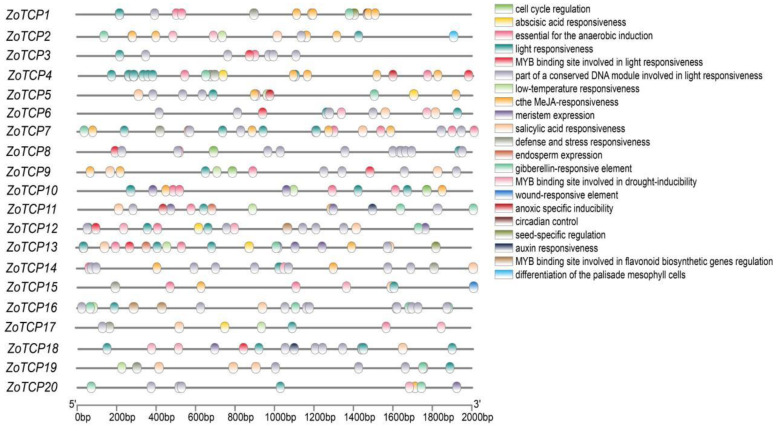
The cis-acting element structure of the *ZoTCP* promoter region; different colors represent different elements.

In order to investigate the role of the *TCP* gene family in ginger’s response to abiotic stress, we performed a comprehensive analysis. Firstly, we extracted the 2000 bp sequence upstream of 20 *ZoTCP* gene promoters using TBtools. Subsequently, we analyzed the cis-acting elements using plantcare. The results of our analysis (Figure 6) revealed the presence of diverse hormone and stress response elements, as well as light response elements. Among the identified cis-acting elements, we focused on 21 elements that exhibited effective expression. Hormone response elements included abscisic acid response element (abscisic acid responsiveness), salicylic acid response element (salicylic acid responsiveness), gibberellin response element (gibberellin responsiveness), auxin response element (auxin responsiveness), and MeJA response element. Stress response elements predominantly consisted of cell cycle regulation response element (cell cycle regulation), anaerobic induction element (anaerobic induction), hypoxia-specific inducibility element (anoxic-specific inducibility), low-temperature responsiveness element (low-temperature responsiveness), and defense and stress responsiveness element (defense and stress responsiveness). Additionally, we identified MYB binding sites involved in drought-inducibility and wound-responsive elements. Furthermore, three types of light responsiveness elements, including MYB light response binding sites and DNA modules related to light response, were found to be the most abundant elements in the promoter region of *ZoTCP*. Notably, only the promoter region of *ZoTCP8* lacked this element. Moreover, our analysis uncovered several other elements related to physiological growth, such as meristem expression element, endosperm expression element, circadian control element, and seed-specific regulation element. These elements played roles in palisade mesophyll cell differentiation and regulation of flavonoid biosynthetic genes.

### 2.7. ZoTCP Gene Replication Events and Collinearity Analysis

**Figure 7:**
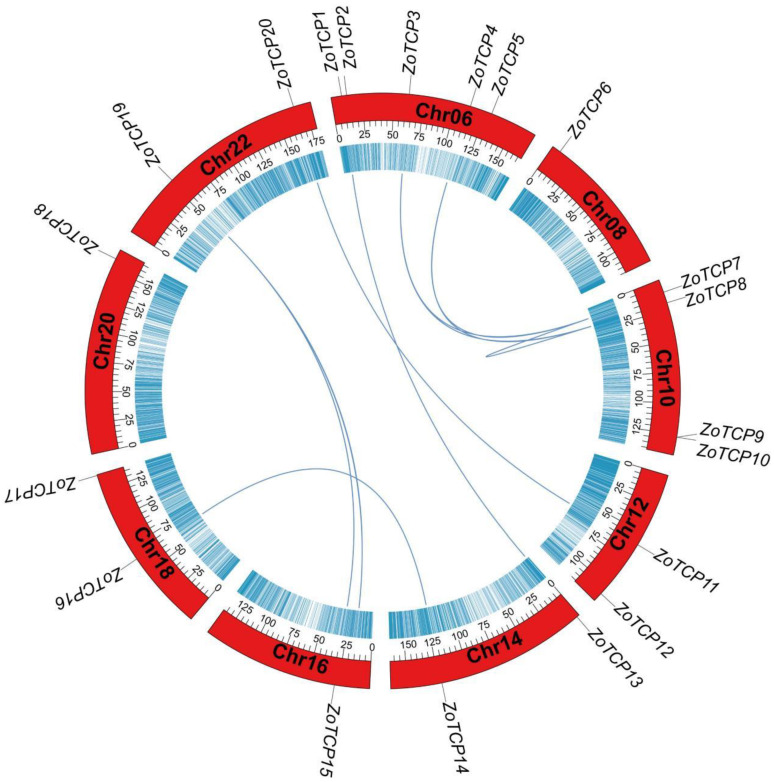
*ZoTCP* genome location and gene repeat fragments; the blue line represents 9 pairs of repeat gene fragments.

The collinearity analysis of 20 *TCP* family genes in ginger revealed no instances of tandem replication events. However, there were 12 *ZoTCP* genes that exhibited fragment replication events, suggesting that fragment replication may be the primary mechanism for amplifying the *TCP* gene family in ginger. These fragment replication events occurred on chromosomes other than Chr.08 and Chr.20, specifically, on *ZoTCP19* and Chr.16. Among the *ZoTCP* genes, nine pairs showed replication events: *ZoTCP16*/*ZoTCP14*, *ZoTCP19*/*ZoTCP15*, *ZoTCP20*/*ZoTCP11*, *ZoTCP1*/*ZoTCP13*, *ZoTCP7*/*ZoTCP8*, *ZoTCP3*/*ZoTCP8*, *ZoTCP3*/*ZoTCP7*, *ZoTCP4*/*ZoTCP8*, and *ZoTCP4*/*ZoTCP7* (Figure 7). Replication events were also observed in other genes located on *ZoTCP19* and Chr.16.

**Figure 8:**
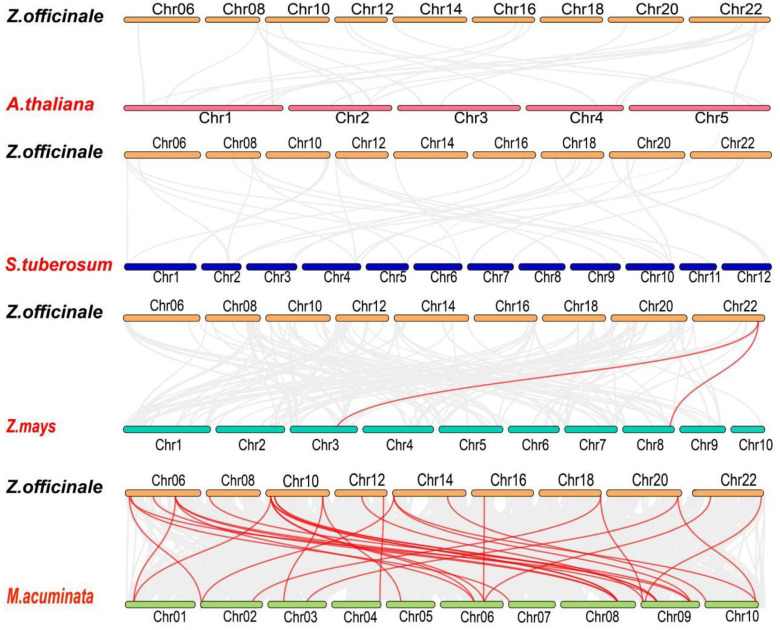
*Z. officinale*(ginger) and *A. thaliana*(arabidopsis), *Z. mays*(corn), *S. tuberosum*(potato), *M. acuminata* (wild banana) *TCP* gene collinearity between the relationship; the red line represents the *TCP* genes having a correlation between the relationship. The red words represents other species that used to compare collinearity with ginger.

When comparing the gene collinearity between ginger *TCP* genes and representative plants such as *Arabidopsis thaliana*, potato, wild banana, and corn, we found that there were no collinearity pairs between ginger and the dicotyledonous plants *Arabidopsis thaliana* and potato. However, there were two collinearity pairs with the small fruit wild banana and 31 collinearity pairs with corn (Figure 8). The absence of gene collinearity between ginger *TCP* genes and dicotyledonous plants suggests that monocotyledonous and dicotyledonous plants may have distinct evolutionary branches. This could be attributed to long-term evolution and environmental pressures, leading to variations in sequence structure similarity between their genomes. Interestingly, we observed 31 gene pairs between ginger and maize, and one gene (*ZoTCP4*) showed collinearity with multiple genes, including *ZoTCP18* and *ZoTCP13*, which are homologous to three genes in Musa acuminata. These findings suggest that *TCP* genes play crucial roles in ginger’s evolution and gene regulation, exerting diverse effects on physiological activities. Additionally, they highlight the higher homology and conservation of *TCP* genes among monocotyledonous plants.

### 2.8. Expression Analysis of ZoTCP in Different Tissues of Ginger

**Figure 9:**
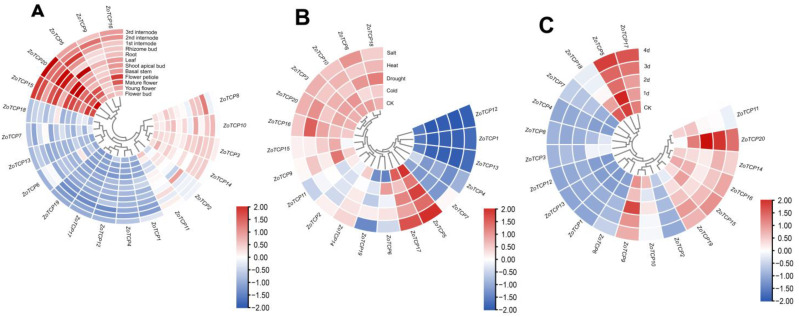
(**A**) The expression pattern of *ZoTCP* in 12 tissues. (**B**) The expression of *ZoTCP* under four abiotic stresses of drought, heat, low temperature, and salt. (**C**) The expression of *ZoTCP* under high temperature and intense light stress.

To explore the biological functions of the *ZoTCP* gene family and understand their role in the growth and development of ginger, we conducted an analysis of expression patterns using transcriptome data from 12 ginger tissues, including roots, stems, leaves, and flowers, across 4 different developmental stages. The expression heat map (Figure 9A) was constructed based on RNA-seq data to visualize the expression profiles of *ZoTCP* genes. The heat map revealed significant variations in the expression of different *ZoTCP* gene family members across various ginger tissues and organs. Most members of the Class I subfamily exhibited high expression levels in all 12 tissues/organs. Specifically, eight genes (66%) belonging to the PCF branch (*ZoTCP2/9/10/11/14/15/16/20*) showed consistently high expression levels across all tissues and organs. This suggests that these genes play crucial roles throughout the growth and development cycle of ginger, including the development of reproductive organs such as flowers. Notably, *ZoTCP15*/*ZoTCP16*/*ZoTCP20* displayed a stepwise expression pattern during different stages of flower development, with high overall expression levels. A similar expression pattern was observed in basal stems and three internodes. In contrast, Class II members generally exhibited low expression levels, except for *ZoTCP5*. Among these, *ZoTCP3*/*ZoTCP5*/*ZoTCP7*/*ZoTCP8* showed moderate expression levels, specifically, in rhizome buds and internode tissues, indicating their involvement in the growth and development of ginger stems. Some genes, such as *ZoTCP1*/*ZoTCP6*/*ZoTCP12*/*ZoTCP19*, exhibited low or undetectable expression levels in various tissues and organs. It is speculated that these genes may either be pseudogenes or possess unique temporal or spatial expression patterns not captured by our data.

### 2.9. Analysis of ZoTCP Expression Patterns under Cold, Drought, Heat, Salt, High-Temperature, and Intense Light Stresses

To investigate the potential functions of *ZoTCP* genes under various abiotic stresses, we utilized RNA-seq data to analyze their expression levels in response to low temperature, heat, drought, and salt treatments. Notably, *ZoTCP1*, *ZoTCP4*, *ZoTCP12*, and *ZoTCP13* exhibited minimal or no expression across all four stress treatments. However, the remaining 16 *ZoTCP* genes showed inducible expression patterns in response to at least 1 stress condition. Specifically, 12 genes (*ZoTCP3*, *ZoTCP5*, *ZoTCP6*, *ZoTCP8*, *ZoTCP9*, *ZoTCP10*, *ZoTCP14*, *ZoTCP15*, *ZoTCP16*, *ZoTCP17*, *ZoTCP18*, *ZoTCP20*) were induced by low temperature, while 12 genes (*ZoTCP3*, *ZoTCP5*, *ZoTCP8*, *ZoTCP9*, *ZoTCP10*, *ZoTCP11*, *ZoTCP14*, *ZoTCP15*, *ZoTCP16*, *ZoTCP17*, *ZoTCP18*, *ZoTCP20*) were induced by drought stress. Moreover, 13 genes (*ZoTCP3*, *ZoTCP5*, *ZoTCP6*, *ZoTCP8*, *ZoTCP9*, *ZoTCP10*, *ZoTCP14*, *ZoTCP15*, *ZoTCP16*, *ZoTCP17*, *ZoTCP18*, *ZoTCP19*, *ZoTCP20*) responded to heat stress. Interestingly, under salt stress, 13 genes (*ZoTCP2*, *ZoTCP3*, *ZoTCP5*, *ZoTCP7*, *ZoTCP8*, *ZoTCP9*, *ZoTCP10*, *ZoTCP14*, *ZoTCP15*, *ZoTCP16*, *ZoTCP17*, *ZoTCP18*, *ZoTCP20*) exhibited induction (Figure 9B). Thus, there were equal numbers of genes induced by low temperature and drought, as well as by salt and heat stress. These findings indicate the widespread involvement of *ZoTCP* genes in abiotic stress responses, while also highlighting expression differences among them. Meanwhile, under high-temperature and intense light stress, 11 *ZoTCP* genes were found to be induced (Figure 9C). These genes included *ZoTCP5*, *ZoTCP9*, *ZoTCP10*, *ZoTCP11*, *ZoTCP14*, *ZoTCP15*, *ZoTCP16*, *ZoTCP17*, *ZoTCP18*, *ZoTCP19*, and *ZoTCP20*. Among them, *ZoTCP9* and *ZoTCP20* reached their peak expression on the second day after treatment and then began to decline. *ZoTCP5* exhibited its highest expression level on the first day after treatment. *ZoTCP17* reached its peak on the fourth day, while *ZoTCP14* and *ZoTCP19* reached their peak expression levels on the third day after exposure to high temperature and intense light. These expression patterns suggest that the expression of these *ZoTCP* genes is time-specific under high-temperature and intense light conditions.

**Figure 10:**
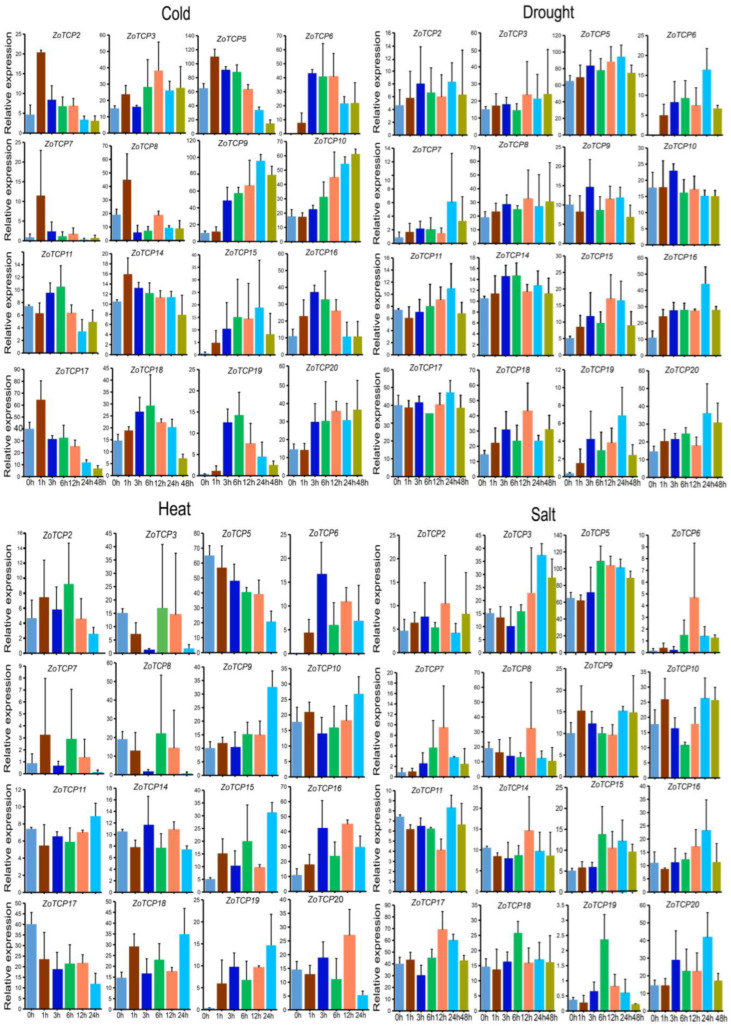
The expression of *ZoTCP* genes under abiotic stress was detected by qRT-PCR; data ae normalized to *TUB-2* gene and the vertical bar indicates the standard deviation.

Furthermore, we performed qRT-PCR analysis to validate the expression levels of these 16 *ZoTCP* genes at different time points after exposure to low temperature, heat, drought, and salt stress. The results (Figure 10) confirmed significant induction of these 16 *ZoTCP* genes under abiotic stress, with varying expression patterns over time. Notably, the expression levels of these genes were up-regulated under drought, low temperature, and salt stress, reaching a peak at specific time points. However, under heat treatment, the expression levels of *ZoTCP5* and *ZoTCP17* exhibited a downward trend (Figure 10). These observations, along with the stress expression pattern data, suggest differential expression of *ZoTCP* genes under various stress conditions and at different time intervals. Specifically, the response rate of these genes to low temperature stress was faster compared to the other stress conditions, with peak expression levels achieved within 1–6 h post-treatment.

## 3. Discussion

Currently, with the availability of genome-wide sequencing data for various plant species, gene family identification has become more accessible. This has enabled the identification of *TCP* genes on a genome-wide scale in numerous species including Arabidopsis, tomato, maize, watermelon, and others. The *TCP* transcription factor family, as a distinct class of transcription factors, has been found to be involved in diverse biological processes such as plant growth, development, and stress response. However, despite the nutritional and medicinal significance of ginger, a commonly consumed and valued plant, there is limited knowledge about its *TCP* gene family and its potential involvement in ginger’s response to adverse conditions. To address this gap, the present study employed bioinformatics analysis to identify 20 *ZoTCP* genes within the *TCP* gene family of ginger. The investigation further involved a comprehensive analysis of protein characteristics, chromosome localization, evolutionary relationships, gene structure, conserved motifs, inter-species evolutionary relationships, cis-acting elements, gene replication events, tissue-specific expression patterns, and expression responses to abiotic stress among *ZoTCP* family members. This thorough examination serves as a foundational step towards understanding the role of the *TCP* gene family in ginger’s growth, development, and response to abiotic stress conditions. Such knowledge holds promise for future research endeavors in this area.

The identified 20 *ZoTCP* genes in this study exhibit a highly conserved *TCP* domain (bHLH) in their corresponding protein sequences, known as Motif1. This suggests the potential for *ZoTCP* to function as DNA-binding protein [14]. Chromosome mapping (Figure 1) revealed that the *ZoTCP* gene family members are distributed irregularly, unevenly, and dispersedly across nine chromosomes. Phylogenetic analysis (Figure 2) and protein sequence alignment (Figure 3) demonstrated that the 20 *ZoTCP* members could be classified into two main subfamilies (Class I and Class II) and three subfamilies (PCF/CIN/CYC/TB1), consistent with previous studies in other species [13]. The phylogenetic tree indicated that each subgroup contained *TCP* genes from ginger, Arabidopsis thaliana, and rice, suggesting the existence of these *TCP* genes in their common ancestors and their inheritance in subsequent generations, forming a shared genetic basis. Furthermore, the distribution ratio of *TCP* genes within each subgroup was biased towards dicotyledonous plants, implying that *TCP* genes underwent expansion from a common ancestor before angiosperm evolution and speciation [38]. Additionally, *ZoTCP* members within the same subfamily exhibited similar exon-intron structures and conserved motifs (Figure 4), further supporting the close evolutionary relationship among *ZoTCP* genes.

Different species exhibit variations in the number of *TCP* family members. For instance, *Arabidopsis thaliana* [39], maize (*Zea mays*) [40], soybean (*Glycine max*) [41], strawberry (*Fragaria ananassa*) [42], and grape (*Vitis vinifera*) [43] possess 24, 46, 54, 19, and 17 *TCP* gene family members, respectively. In this study, a total of 20 *TCP* genes were identified in ginger. The differences in *TCP* gene family numbers among species can be attributed to genomic replication events during species evolution [44]. To investigate the amplification of the *TCP* gene family in ginger, this study analyzed *ZoTCP* replication events. Analysis results (Figure 7) revealed that *ZoTCP* primarily underwent fragmentary replication events, with 12 *ZoTCP* genes involved in such events. Previous studies have demonstrated that many gene families tend to undergo amplification via fragmentary replication and tandem replication. These events serve as crucial driving factors for the functional diversity and evolution of gene families [45,46,47]. These findings align with those observed in Arabidopsis and rice, suggesting a similar replication pattern for the *TCP* gene family in plant genomes [48]. Furthermore, studies have indicated that repetitive genes are expressed in different tissues or organs, implying that these repetitive genes possess specific or redundant cellular functions throughout ginger’s growth and development [49]. For instance, *ZoTCP20*/*ZoTCP11* exhibited differential expression patterns. *ZoTCP20* displayed high expression levels in rhizome buds, flower buds, leaf apical buds, basal stems, third internodes, and roots, while *ZoTCP11* showed no expression in the first internode and mature flowers, with low expression levels in the remaining 12 tissues and organs (Figure 9A). However, analysis of gene structure revealed identical motif composition (Motif1, 2, 3) and positions between these genes, along with eight pairs of repeated gene motifs that were also exactly the same (Figure 4B). This suggests that differences in expression patterns among repeated genes may arise from gene mutations during the replication process or variations in upstream regulatory mechanisms [50], resulting in functional divergence.

Analyzing gene structure and conserved motifs is a vital approach for comprehending gene function and classification, ensuring research integrity. The investigation of gene structure reveals that most *ZoTCPs* within the same subfamily possess identical exon–intron structures. The positional information of introns and exons in the genome serves as crucial evidence for establishing their evolutionary relationship [51]. Furthermore, the analysis of conserved motifs demonstrates that nearly all members exhibit Motif1 (Figure 4B), and genes with similar motif arrangements are grouped together in accordance with evolutionary classification [38]. Taken together, these distinctive gene structures exert a significant impact on protein function.

The analysis of an interspecific phylogenetic tree revealed the presence of *TCP* proteins in each subfamily among five different species (Figure 5A). Furthermore, a conserved motif analysis (Figure 5B) indicated the existence of a common motif (Motif1) in *TCP* genes across all species, suggesting the early emergence of *TCP* genes in these representative plants prior to differentiation [38]. Collinearity analysis demonstrated no observed collinearity relationship between *ZoTCP* and dicotyledonous plants. However, a collinearity relationship was detected between *ZoTCP* and monocotyledonous plants, specifically, maize and small fruit wild banana. This finding suggests that *TCP* genes undergo functional and expression divergence during subsequent differentiation, likely due to differences in species morphology, growth environment, and adaptability.

Extensive research has demonstrated the involvement of *TCP* transcription factors in various biological functions pertaining to plant growth, development, and response to environmental stresses. These functions encompass seedling germination, stamen development, stem branching, leaf development, flower development, and senescence, as well as temperature immunity [23,52,53]. To gain insights into the function of *ZoTCP*, we conducted an analysis of the cis-acting elements within the promoter region of *ZoTCP*. The results (Figure 6) revealed the presence of several significant elements, including those involved in light response, hormone response (such as abscisic acid, gibberellin, salicylic acid, MeJA, auxin), stress response (anaerobic, low temperature), meristem and endosperm expression, and MYB-binding drought site response. These findings indicate that the *ZoTCP* gene family not only participates in diverse abiotic stress responses but also regulates ginger’s growth and development by modulating hormone levels [38].

The flowering mechanism of ginger remains unclear, even though *TCP* has been implicated in regulating flowering in model plants like *Arabidopsis thaliana* [54]. In our study, significant expression of *ZoTCP* was observed during the developmental stages of flowers (Figure 9A). For instance, *ZoTCP20* exhibited expression in flower buds, young flowers, and mature inflorescences. *ZoTCP15* showed higher expression in flower buds and young flowers compared to mature inflorescences. Furthermore, *ZoTCP16* displayed higher expression in mature inflorescences and pedicels compared to other tissues. These findings suggest that these three *ZoTCP* genes might be involved in regulating the growth and development of ginger flowers. Additionally, these three *ZoTCPs* belong to the PCF subfamily and share close homology. In Arabidopsis, *AtTCP8* and *AtTCP23*, also members of the PCF subfamily, are key transcription factors involved in regulating flowering [55]. However, there are notable differences between *ZoTCP* and *AtTCP* in their *TCP* motif composition, particularly Motif6 and Motif4 at the N-terminus and C-terminus, respectively. These differences suggest potential functional distinctions and a considerable divergence in homology between the two. Consequently, further experiments are necessary to elucidate the specific role of *ZoTCP* in ginger flowering. As ginger is an economically significant crop, with its rhizome being a valuable component, the enlargement of rhizomes is of primary importance in ginger cultivation. In our study, *ZoTCP15* exhibited high expression in roots, rhizome buds, and second and third internodes, with expression increasing gradually in internodes. *ZoTCP20*, on the other hand, displayed high expression in roots, rhizome buds, and third internodes. Thus, additional experiments are required to verify the regulatory mechanisms of *ZoTCP15* and *ZoTCP20* in the growth and development of ginger rhizomes.

Plant growth, development, and productivity are significantly influenced by abiotic stresses. *TCP* genes play a crucial role in regulating various aspects of plant life. Therefore, it is essential to investigate the potential functions of *TCP* genes under different abiotic stress conditions [31,56]. In our study, we aimed to explore the role of *ZoTCP* genes under abiotic stress by analyzing their regulatory effects during drought, low temperature, heat, and salt treatments using RNA-seq and qRT-PCR techniques. The expression patterns of *ZoTCP* genes exhibited significant variations under stress treatments. Earlier studies have shown that *OsPCF6* and *OsTCP21* impact rice sensitivity to low temperatures. Upregulating these genes through RNA interference (RNAi) increases the accumulation of osmoprotectants like free proline and DREB1/CBF proteins in rice. These osmoprotectants help minimize oxidative damage during non-biological stress conditions. Furthermore, reducing the activity of reactive oxygen species (ROS) scavengers by downregulating *OsPCF6* and *OsTCP21* enhances cold stress tolerance in plants [31]. Based on the expression patterns (Figure 9B) and qRT-PCR results (Figure 10), we found that *ZoTCP9* and *ZoTCP5* were notably induced by cold stress, showing higher expression levels compared to other *ZoTCP* members. Both *ZoTCP9* and *ZoTCP5* contain cis-acting elements related to low-temperature response. However, their expression patterns differed. *ZoTCP5* exhibited peak expression at 1 h after cold induction, followed by a gradual decrease over 48 h. In contrast, *ZoTCP9* showed a gradual increase in expression after cold induction, reaching its peak at 24 h. Furthermore, in the phylogenetic tree analysis, *ZoTCP5* and *OsPCF6* clustered together in the same CIN subfamily branch, while *ZoTCP9* and *OsTCP21* clustered in the same PCF subfamily branch. According to the current understanding of gene function similarity within phylogenetic tree branches across multiple species, it can be inferred that *ZoTCP5* and *ZoTCP9* play vital roles in responding to cold stress. Interestingly, *ZoTCP5* demonstrated high expression levels under all four abiotic stresses: low temperature, heat, drought, and salt. This suggests the potential involvement of *ZoTCP5* in multiple abiotic stress responses and warrants further exploration of its specific functions in these conditions.

## 4. Materials and Methods

### 4.1. Plant Materials and Treatments

The materials used in this study were sourced from *Z. ofcinale* cv. Southwest, which was cultivated in the greenhouse of Chongqing University of Arts and Sciences, Chongqing, China. Two-month-old ginger seedlings in pots were exposed to an outdoor environment under high-temperature and intense light stress for four days of treatment in July. The temperature could reach a maximum of 40 °C, and the light intensity could reach up to 103,833 Lux. Functional leaves of ginger, specifically, the 3rd to 5th unfolded leaves from the top to the base of the stem, were selected for sampling. The leaves of ginger, before being moved to the outdoor environment, were collected at 8:30 a.m. and used as control. For the treatments, leaves were collected at 3:00 p.m. from the first day to the fourth day. After collection, the plant materials were promptly placed in liquid nitrogen at −80 °C for preservation and subsequent analysis. Each sample was prepared with three technical replicates to ensure accuracy.

### 4.2. Identification and Physicochemical Properties Analysis of TCP Gene Family Members in Ginger

The ginger genome data utilized in this study were obtained from our research group’s ginger genome research project. Firstly, we downloaded the standard *TCP* transcription factor HMM (hidden Markov model) file (login number: PF03634) from the Pfam database (http://pfam-legacy.xfam.org/, accessed on 21 June 2023) [57]. Subsequently, the HMMER 3.0 software [58] was employed to search the protein sequence (Supplementary Material File S2: Table S2) library of ginger, and candidate genes with an E-value of 0.0001 were selected. To further refine the selection, SMART (http://smart.embl.de/, accessed on 23 June 2023) was utilized to identify ZoTCP protein sequences, eliminating those that lacked the bHLH motif. Consequently, the gene family responsible for encoding TCP proteins in ginger was identified. To analyze the physical and chemical properties of the processed TCP protein sequences from ginger, tools such as Protparam (https://web.expasy.org/protparam/, accessed on 24 June 2023) were employed. This analysis encompassed parameters such as isoelectric point, molecular weight, and amino acid length [59]. The chromosomal location information of the *ZoTCP* was obtained from its genome annotation file. The TBtoolsv1.123 software (https://github.com/CJ-Chen/TBtools, accessed on 21 June 2023) [60] was utilized to determine and describe the precise position of *ZoTCP* on the chromosome.

### 4.3. Multiple Sequence Alignment and Phylogenetic Tree Construction of Ginger TCP Protein

The BioEdit7.0.9 software was utilized to compare and analyze the amino acid sequence of the TCP protein, including the comparison of conserved domains [61]. Multiple alignments were performed using MEGA11.0.10 software [62] between the protein sequences of the identified *ZoTCP* gene family and TCP gene family sequences obtained from the *Arabidopsis thaliana*, *Oryza sativa*, *Solanum tuberosum*, *Zea mays*, and *Musa acuminata*. These sequences were downloaded from the TFDB database (http://planttfdb.gaolab.org/index.php, accessed on 28 June 2023) [63]. The Neighbor-Joining method with 1000 bootstrap test iterations was employed, and default parameter values were selected for phylogenetic tree construction. Moreover, we utilized the maximum likelihood method to construct a phylogenetic tree to validate the phylogenetic tree generated by the neighbor-joining method. The parameters were set as 80% cutoff, JTT+G amino acid substitution model, and 1000 bootstrap replicates. The resulting phylogenetic tree was visually enhanced using the online tool Evolview (https://www.evolgenius.info/evolview-v2/, accessed on 29 June 2023).

### 4.4. Analysis of TCP Gene Structure and Conserved Elements in Ginger

The gene structure of *ZoTCP* was analyzed using the online tool GSDS (http://gsds.cbi.pku.edu.cn/, accessed on 25 June 2023) [64]. This tool facilitated the examination of the exon and intron structure of ZoTCP. To analyze the *TCP* gene sequence further, the MEME online tool (https://meme-suite.org/meme/doc/meme.html, accessed on 25 June 2023) was employed. During the detection process, 10 motif values were specified, while other default values were selected [65].

### 4.5. Analysis of Cis-Acting Elements of Ginger TCP Gene

The cis-acting elements of the *TCP* gene in ginger were analyzed using the online tool PlantCARE (https://bioinformatics.psb.ugent.be/webtools/plantcare/html/, accessed on 30 June 2023) [66] and the TBtoolsv1.123 software (https://github.com/CJ-Chen/TBtools, accessed on 1 July 2023) [60]. Through this analysis, specific cis-acting elements that had a significant impact on ginger were identified.

### 4.6. Collinearity Analysis

Subsequently, the replication event of *ZoTCP* itself was analyzed. Using the gene sequence file, the gene replication of *ZoTCP* was examined using MCScanX, with the default parameters of TBtools. Furthermore, gene data files for Arabidopsis thaliana, potato, small fruit wild banana, corn, and other species were obtained from the EnsemblGenomes database (https://ensemblgenomes.org/, accessed on 3 July 2023) [67] The TBtoolsv1.123 software was employed to analyze the collinearity between these species’ gene sets and the *ZoTCP* genes, and a collinearity relationship diagram was generated.

### 4.7. ZoTCP Gene Expression and qRT-PCR Analysis

Our research group’s previous transcriptome data of various tissues of ginger and ginger under abiotic stress enabled us to analyze the expression patterns of ZoTCP under different tissue and abiotic stress conditions. Ginger tissues include young flowers, flowers, flower stalks, rhizome buds, flower buds, the first, second, and third internodes and basal stems, apical buds, and roots and leaves. Abiotic stress includes low temperature, heat, drought, and salt stress. Drought stress and salt stress treatments were conducted by irrigating the plants with a 15% PEG6000 solution and a 200 mM sodium chloride solution, respectively. High-temperature stress and low-temperature stress treatments were applied to ginger plants at 40 °C and 4 °C, respectively. Leaf samples of ginger were collected at specific time points of 1 h, 3 h, 6 h, 12 h, 24 h, and 48 h after low-temperature stress, drought stress, and salt stress treatments. The leaf samples were collected at time points of 1 h, 3 h, 6 h, 12 h, and 24 h for the heat treatment. The expression patterns of ZoTCPs were visualized using the HeatMap plugin in the TBtoolsv1.123 software.

Primers for *ZoTCP* members (Table S1) were designed using Primer 5.0 software, and qRT-PCR experiments were conducted to analyze their response to abiotic stress. The ZoTUB2 gene was used as an internal reference control, and relative gene expression levels were evaluated using the 2^−∆∆Ct^ method [68]. All qRT-PCR detections were performed with three biological and technical replicates.

## 5. Conclusions

In this study, 20 *ZoTCPs* were identified from the whole genome of ginger, which were distributed on 9 chromosomes. Phylogenetic analysis divided them into two subgroups: Class I and Class II. We discovered that the 20 *ZoTCP* genes consist of 9 pairs of repetitive genes and are distributed across different positions on 7 chromosomes. Fragment duplication serves as the primary mechanism for *ZoTCP* gene amplification. The expression patterns of *ZoTCPs* in various tissues and organs indicate their significant role in the growth and development of ginger. Additionally, our promoter analysis revealed that the *ZoTCP* promoter region generally contains cis-acting elements (such as low-temperature responsiveness, defense, and stress responsiveness) that respond to abiotic stress. By studying the evolutionary relationship and conducting collinearity analysis among different species, we gained insights into the evolutionary characteristics of ginger and made predictions regarding related gene functions. Our expression analysis of *ZoTCP* under different abiotic stresses suggests that *ZoTCP5* and *ZoTCP9*, which are homologous to *OsPCF6* and *OsTCP21*, respectively, may play a role in ginger’s response to low-temperature stress. Furthermore, these genes are also associated with stress-related cis-acting elements present in their promoters. In conclusion, these results hold significant importance for the analysis of *ZoTCP* genes and provide a theoretical foundation for the rational cultivation of ginger, stress resistance breeding, and the future verification of *ZoTCP* gene functions. Therefore, further investigation into the response mechanism of *ZoTCP* involved in ginger’s abiotic stress is necessary.

## Figures and Tables

**Table 1:** The information of *TCP* family genes in *Zingiber officinale*.

Gene Name	Gene ID	Chromosome	Localization (bp)	Animo Acid Number	Molecular Weight (kD)	PI
*ZoTCP*1	ZOFF_071857	Chr.06	8,518,591–8,519,676 (+)	361	38.71	6.72
*ZoTCP*2	ZOFF_071376	Chr.06	12,551,671–12,552,432 (+)	253	25.82	9.16
*ZoTCP*3	ZOFF_058948	Chr.06	62,385,964–62,387,566 (−)	464	49.34	9.38
*ZoTCP*4	ZOFF_070666	Chr.06	111,911,935–111,913,806 (−)	565	61.24	6.73
*ZoTCP*5	ZOFF_055205	Chr.06	129,782,255–129,783,586 (−)	443	48.45	7.00
*ZoTCP*6	ZOFF_035032	Chr.08	6,982,140–6,983,267 (−)	375	39.81	8.47
*ZoTCP*7	ZOFF_033278	Chr.10	10,437,715–10,439,388 (+)	501	54.58	6.68
*ZoTCP*8	ZOFF_009476	Chr.10	19,251,872–19,254,729 (−)	622	66.94	8.24
*ZoTCP*9	ZOFF_010045	Chr.10	127,022,793–127,023,695 (−)	300	30.89	5.77
*ZoTCP*10	ZOFF_009968	Chr.10	127,027,323–127,028,273 (−)	316	32.45	6.01
*ZoTCP*11	ZOFF_005937	Chr.12	59,942,137–59,948,621 (+)	323	33.88	7.21
*ZoTCP*12	ZOFF_075892	Chr.12	108,521,821–108,522,456 (+)	211	23.49	8.83
ZoTCP13	ZOFF_038801	Chr.14	122,725,201–122,726,148 (−)	364	39.35	7.17
*ZoTCP*14	ZOFF_035356	Chr.14	2,292,226–2,293,320 (−)	315	32.74	7.90
*ZoTCP*15	ZOFF_068839	Chr.16	28,238,917–28,239,843 (+)	308	33.20	6.87
*ZoTCP*16	ZOFF_008749	Chr.18	64,415,581–64,416,537 (+)	318	33.08	6.67
*ZoTCP*17	ZOFF_057997	Chr.18	137,811,656–137,812,966 (+)	436	47.31	8.95
*ZoTCP*18	ZOFF_000424	Chr.20	159,161,052–159,166,863 (+)	330	37.05	8.63
*ZoTCP*19	ZOFF_047522	Chr.22	52,215,302–52,229,031 (−)	713	78.70	8.34
*ZoTCP*20	ZOFF_003424	Chr.22	165,907,234–165,908,193 (−)	319	33.27	6.84

## Data Availability

The amino acid sequence of ZoTCPs are available in Supplementary Material File S2. The relevant datasets used and/or analyzed during the current study can be made available upon request to the corresponding author.

## Supplementary Materials

The following supporting information can be downloaded at: https://www.mdpi.com/article/10.3390/plants12193389/s1, Table S1: The primer sequences for qRT-PCR used in this study. Supplementary Material File S2: Figure S1: Phylogenetic tree constructed by maximum likelihood method. Table S2: Ginger TCP protein sequence information. Supplementary Material File S3: Figure S2: The motif structure of ZoTCP protein contains 10 Motifs. Figure S3: Composition of TCP protein motifs in five different plants.
