# Supplementary figures and images for: Genome-Wide Identification and Expression Analysis of the TCP Gene Family Related to Developmental and Abiotic Stress in Ginger
Source: Plants (Basel). 2023 Sep 26;12(19):3389. doi: 10.3390/plants12193389 (PMC10574737; doi:10.3390/plants12193389)

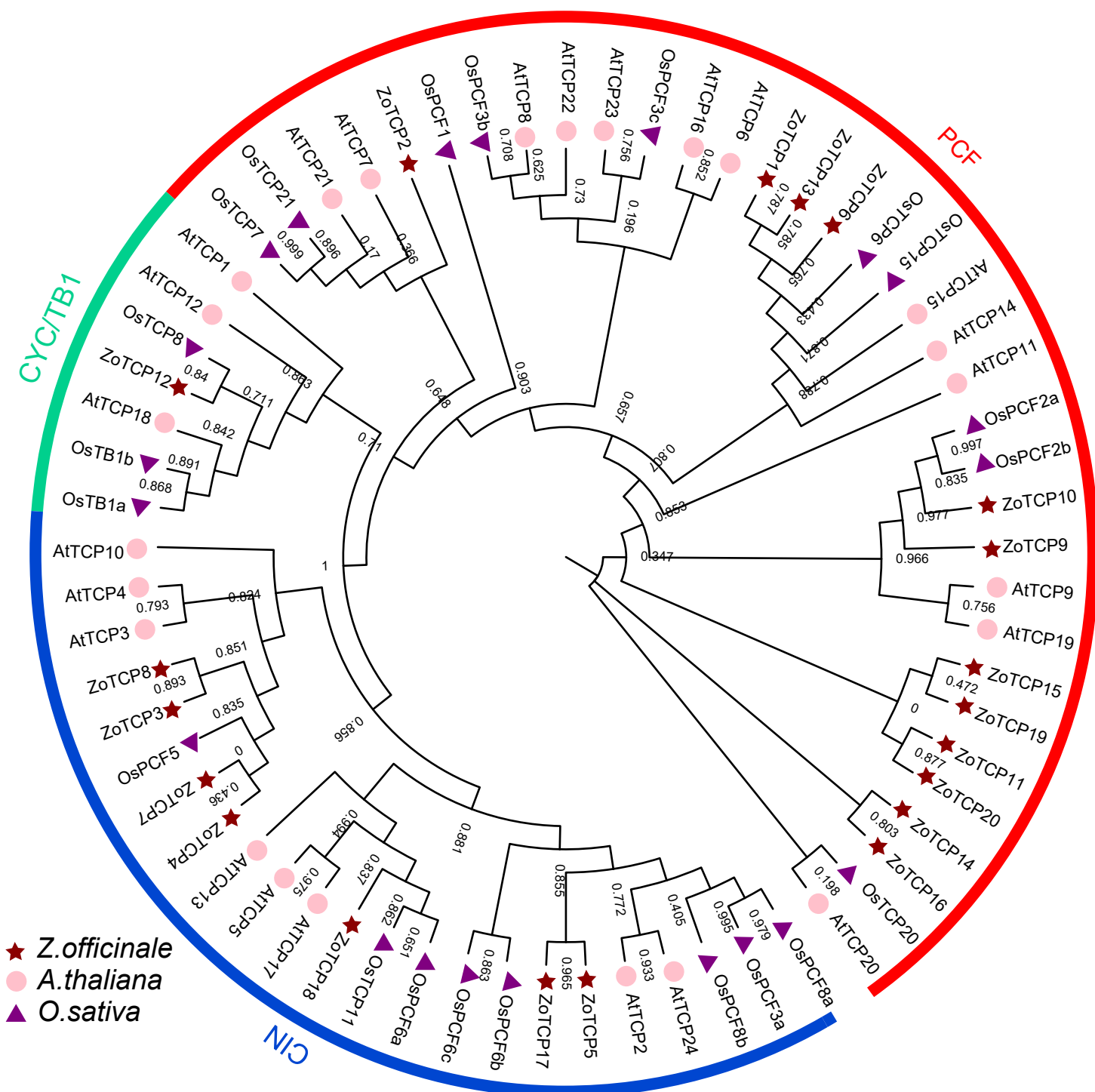

Supplement: Supplementary file 1 [file plants-12-03389-s001.zip › plants-2581850-supplementary/All Supplementary Material/Supplementary Material File S2/Fig. S1 Phylogenetic tree constructed by maximum likelihood method.pdf]

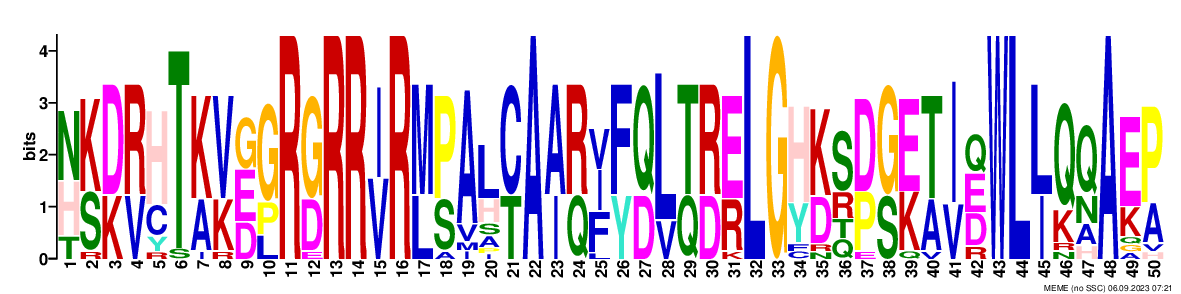
Motif1


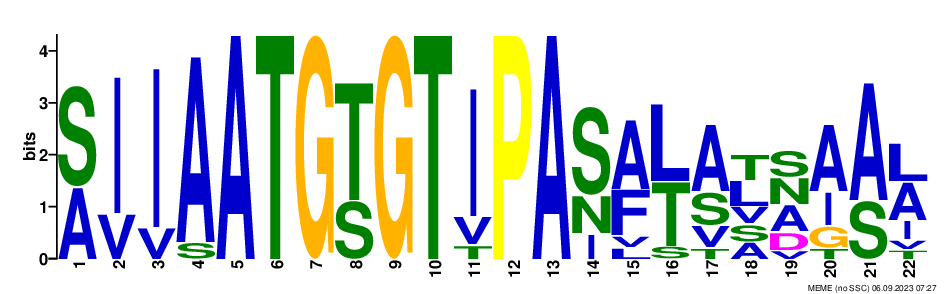
Motif2

Motif3


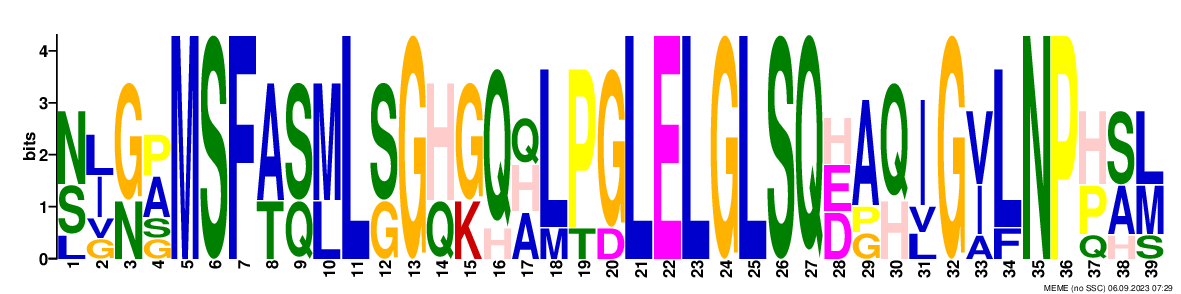


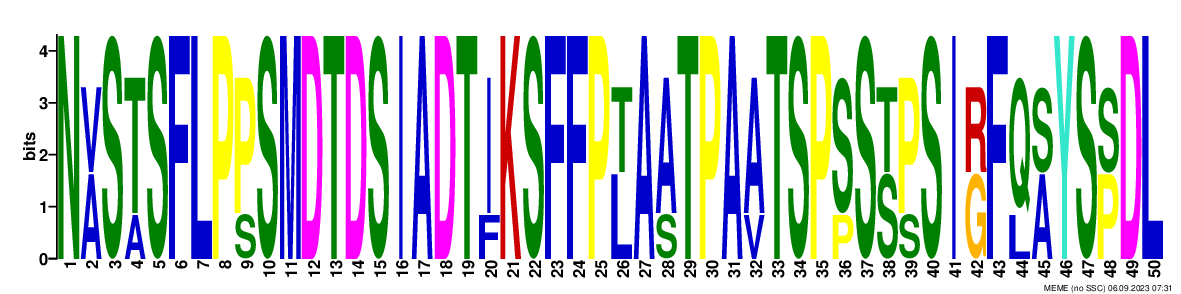
Motif4

Motif5


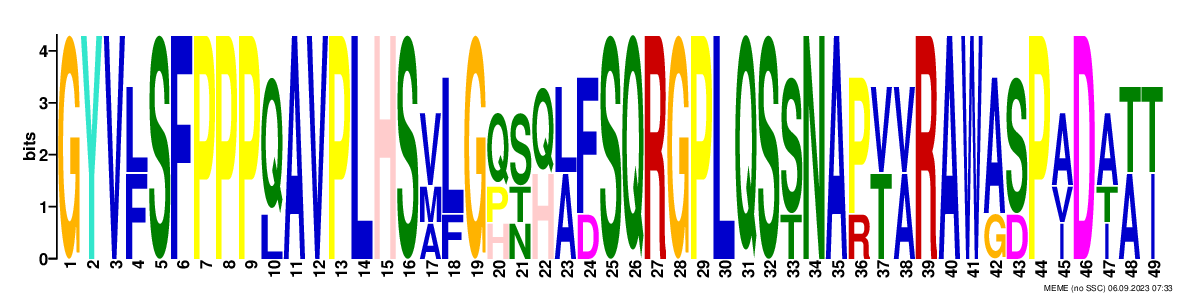


Motif6


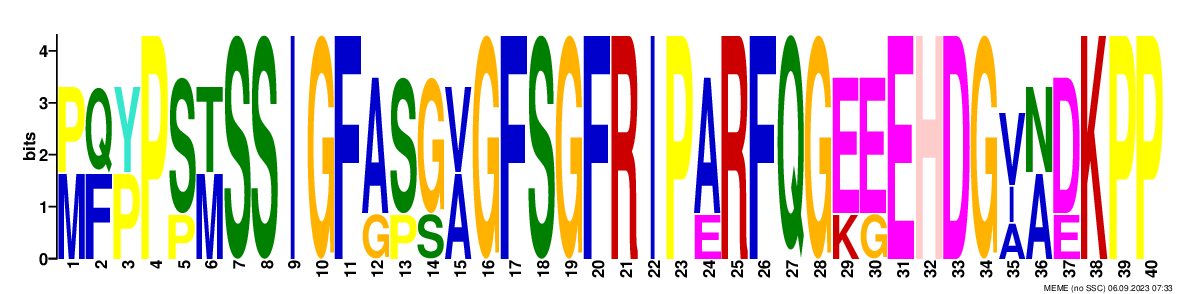


Motif7


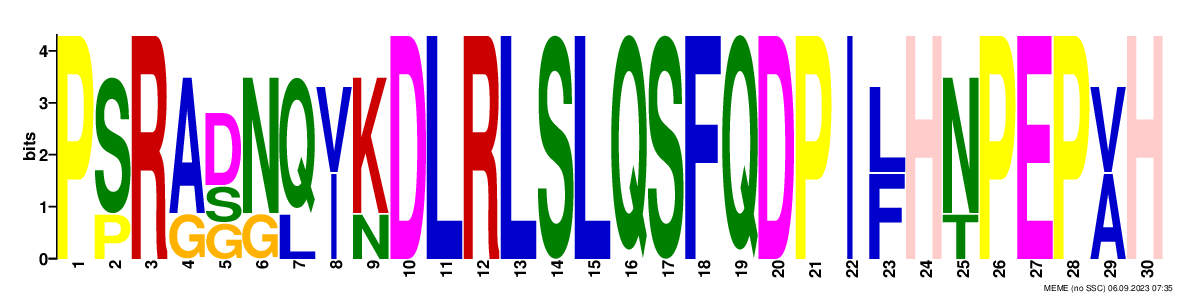


Motif8


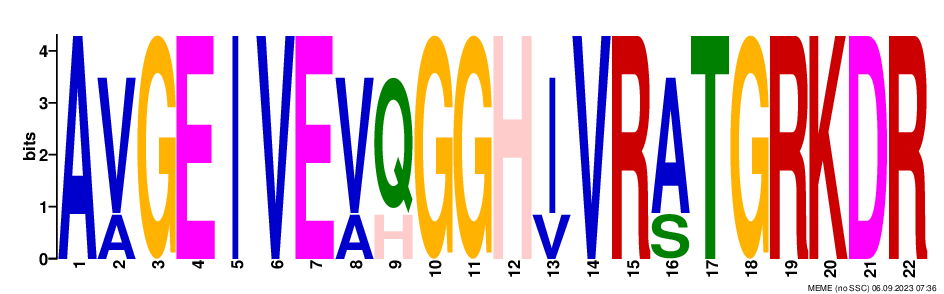


Motif9


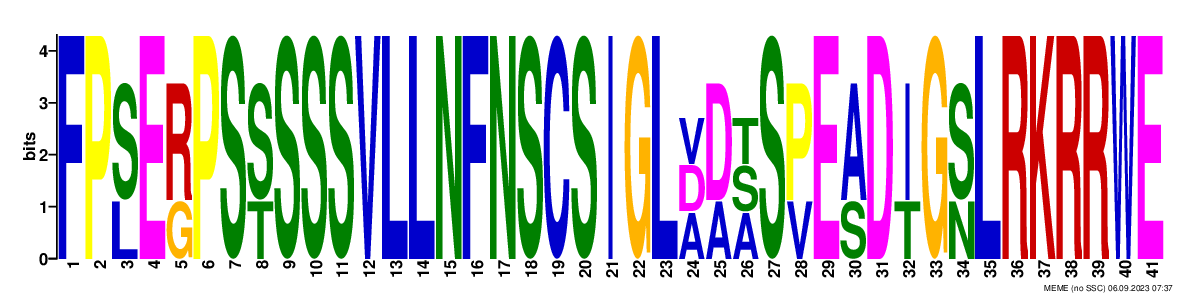


Motif10


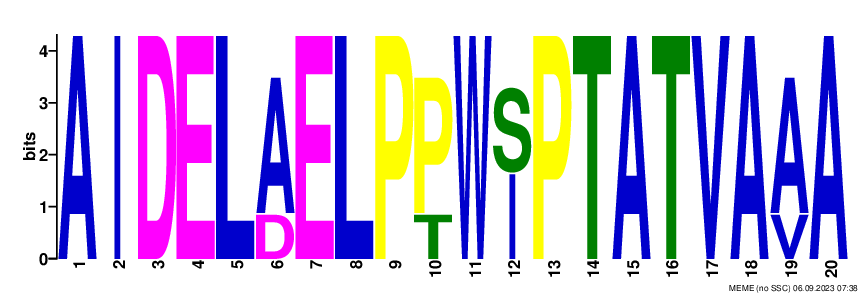

Supplement: Supplementary file 1 [file plants-12-03389-s001.zip › plants-2581850-supplementary/All Supplementary Material/Supplementary Material File S3/Figure S2.The motif structure of ZoTCP protein contains 10 Motifs.docx]

Motif1


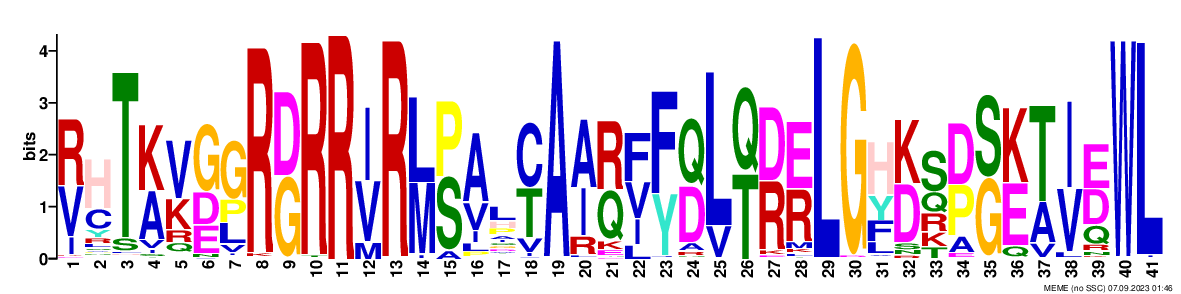


Motif2


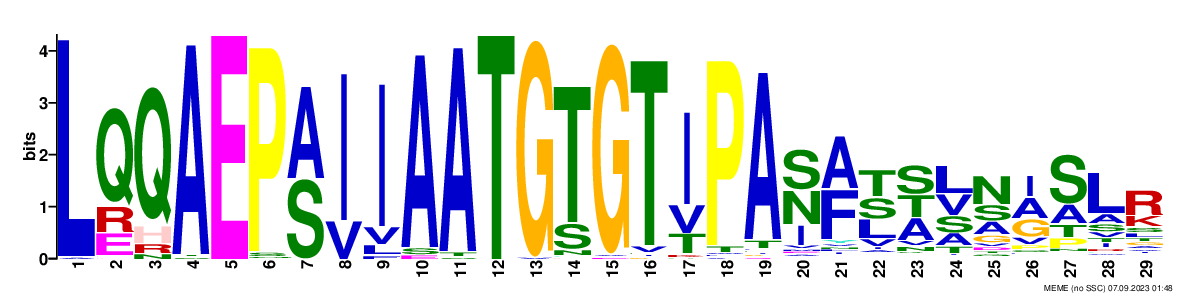


Motif3


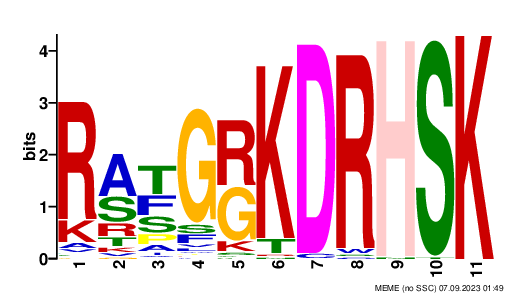


Motif4


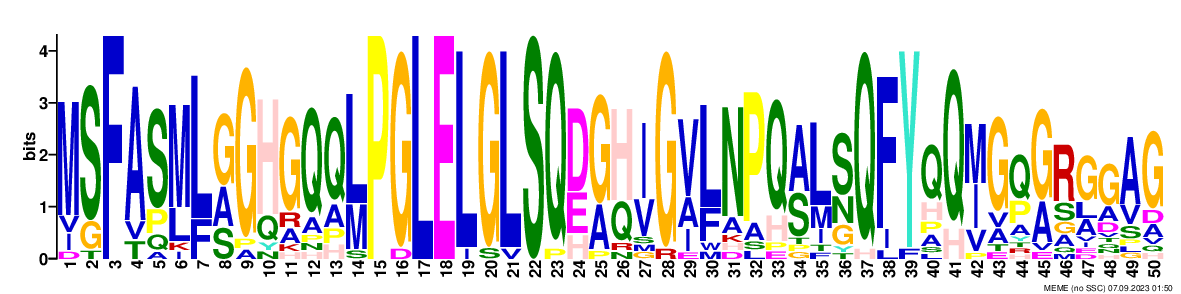


Motif5


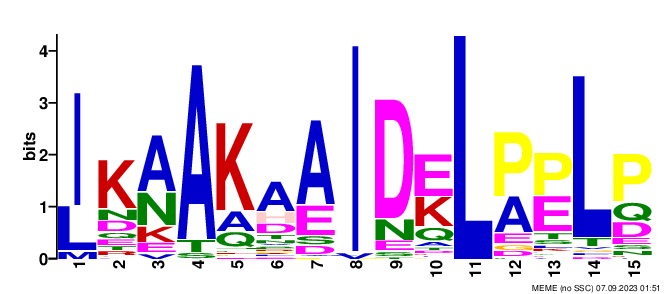


Motif6


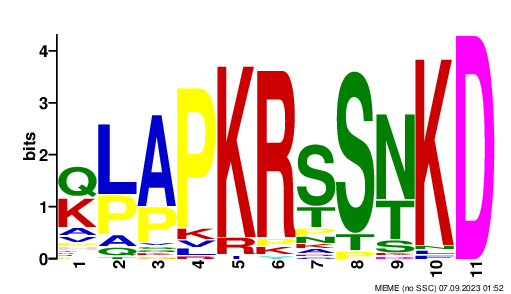


Motif7


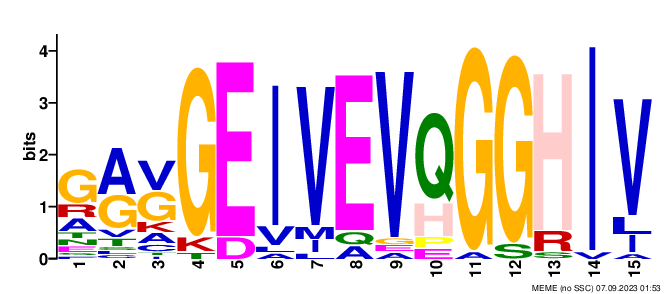


Motif8


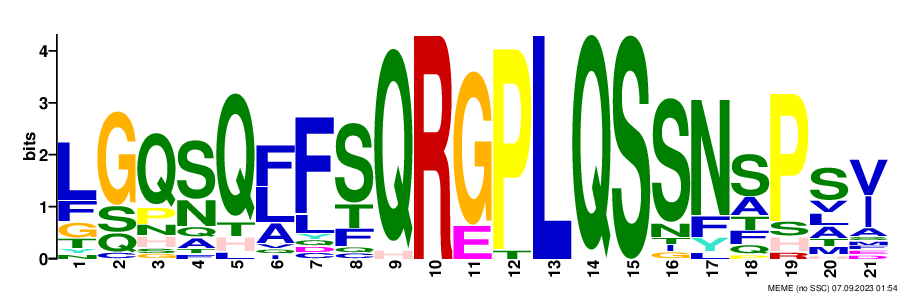


Motif9


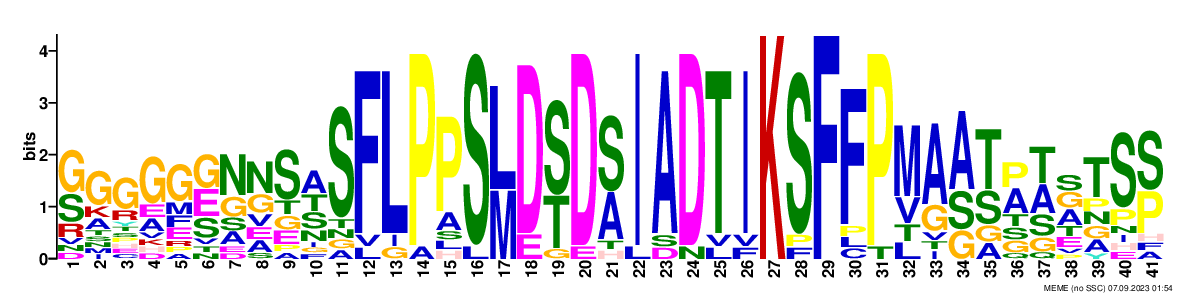


Motif10


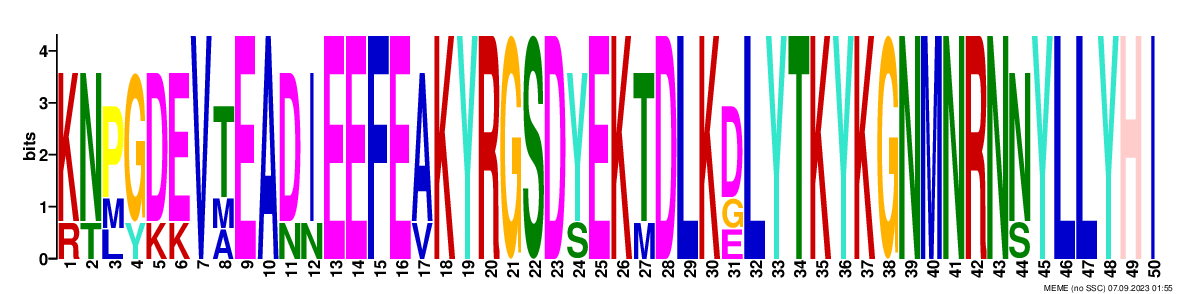

Supplement: Supplementary file 1 [file plants-12-03389-s001.zip › plants-2581850-supplementary/All Supplementary Material/Supplementary Material File S3/Figure S3. Composition of TCP protein motifs in five different plants.docx]
